# Editorial: Sleep disturbances in Parkinson's disease

**DOI:** 10.3389/fnins.2023.1133296

**Published:** 2023-02-02

**Authors:** Maria Salsone, Luigi Ferini-Strambi

**Affiliations:** ^1^Institute of Molecular Bioimaging and Physiology, National Research Council, Milan, Italy; ^2^Sleep Disorders Center, Division of Neuroscience, San Raffaele Scientific Institute, Milan, Italy; ^3^Department of Clinical Neurosciences, Neurology, Vita-Salute San Raffaele University, Milan, Italy

**Keywords:** sleep disturbances, Parkinson's disease, magnetic resonance imaging (MRI), DAT-SPECT imaging, functional MRI

It is well-established that sleep disorders (SDs) may be present in the clinical picture of Parkinson's disease (PD). This could be related to the following aspects: (i) SDs may share PD pathogenesis, representing a prodromal phase of the disease, as demonstrated by REM sleep behavior disorder (RBD); (ii) SDs may be caused by primary PD neurodegeneration, which is the case for excessive daytime sleepiness (EDS) with abnormalities of the brainstem sleep/wake-promoting nuclei and/or respiratory centers; and finally (iii), advanced age and the presence of systemic comorbidities often reported in PD represent potential risk factors for the development of sleep breathing disorders, such as obstructive sleep apnea (OSA). Although there is solid evidence supporting the strict relationship between SDs and PD, novel intriguing questions emerge in this wide scenario. First, it is interesting to note that not all PD patients manifest SDs, and not all present these in the same temporal sequence (SD may precede PD onset, be concomitant, or manifest after the typical motor manifestation of the disease). Second, it is also well-documented that the occurrence of SDs as a core feature of PD may be associated with cognitive impairment shifting the clinical phenotype toward one that is at higher risk of dementia. In this context, the contribution of specific SDs in the neural circuits involved in the pathogenesis of PD-cognitive decline is debatable. The spectrum of cognitive impairment in PD ranges from mild cognitive impairment to overt dementia. Two main profiles, often coexistent, have been identified: (i) a relatively slow decline, related to dopaminergic dysfunctions and clinically characterized by frontostriatal executive deficits, and (ii) a more rapid decline caused by the degeneration of cholinergic projection fibers in the basal forebrain and posterior cortical alterations, clinically manifested by visuospatial and memory deficits (Pagonabarraga and Kulisevsky, 2012). The early identification of PD patients at high risk of developing cognitive decline is crucial not only for the prognosis but also for the treatment of potential SD risk factors. Therefore, the current Research Topic aims to answer all these interesting questions defining the global cognitive trajectories that, from SDs, lead to cognitive decline in PD.

The first research article is focused on the characterization of the neurocognitive circuits in patients with PD associated with EDS (PD-EDS) (Wang et al.). The topic is of interest, as EDS is a common feature of PD, affecting up to 60% of patients, presents from the initial stage of the disease, and impairs cognitive performance. PD-EDS patients may have altered global cognitive functioning and attention deficits at baseline, with a greater rate of worsening Frontal Battery scores over time (Chan et al., 2020). Supporting the clinical findings, widespread cortical and subcortical alterations, such as atrophy of the frontal lobe (Kato et al., 2012), hypoperfusion in associative cortices (Matsui et al., 2006), and reduced caudate DAT uptake, as shown by DAT-SPECT imaging (Yousaf et al., 2018), have been found in PD-EDS patients. Overall, these findings suggest that PD-EDS patients may have a frontostriatal cognitive impairment over time.

The research article that we present here fits perfectly with this context. It is a resting state functional magnetic resonance imaging (f-MRI) study focused on the potential brain changes occurring in PD-EDS (Wang et al.). The authors investigated the neural activity changes in male PD patients with and without EDS, and in controls. Their protocol included analysis of neural activity at two levels: the amplitude of low-frequency fluctuations (ALFF) at the local level, and functional connectivity (FC) at the network level. Their main results demonstrated that (i) PD-EDS patients showed abnormal ALFF in the pons and frontal areas compared with the controls, and (ii) PD-EDS patients exhibited altered neural activity, with decreased ALFF in the cingulate cortex and reduced FC of the cingulate cortex and precuneus compared with those without EDS. The first result supports the hypothesis that the functional wake-promoting pathway deficits might cause hypersomnia in PD. Indeed, as reported by the same authors, the pons contain the wake-promoting neuronal populations that can activate the thalamus, which in turn arouses the neurons in the cerebral cortex. The second result has a broader meaning. It is interesting to note that the brain structures with reduced FC, cingulate cortex, and precuneus not only represent two crucial nodes of the default mode network (DMN) but also key structures involved in the circuits underlying the pathogenetic process in Alzheimer's disease. Although preliminary, these findings may significantly contribute to better defining the neural mechanisms underlying the cognitive impairment related to EDS in PD patients.

The second research article is focused on the impact of SDs, such as insomnia, RBD, and sleep breathing disorders, particularly OSA, on cognitive performance in patients with PD (Hermann et al.). OSA is frequently reported in PD patients (20–60%) and is considered a common comorbidity rather than a consequence of PD neurodegeneration. Among PD-related comorbidities, OSA represents the main risk factor for the development of cognitive decline. Indeed, features related to OSA, including intermittent hypoxia and sleep fragmentation, can accelerate the accumulation of β-Amyloid and Tau-proteins in the brain and independently trigger an Alzheimer's-type neuropathology in PD. Longitudinal investigation reported that PD-OSA showed a more severe deterioration, especially of executive functions at the 4-year follow-up (Meira et al., 2022). Interestingly, CPAP treatment of OSA in PD improved overall non-motor symptoms and global cognitive functions over a 12-month period (Kaminska et al., 2018). Here, we present a clinical investigation perfectly focused on all these questions (Hermann et al.). The authors designed a *post-hoc* analysis of the RaSPar trial, a single-center double-blind baseline-controlled clinical trial conducted by Technische Universität Dresden, Germany. Their protocol included two sections of evaluations: the first section involved clinical and polysomnographic assessments (interviews, questionnaires, and polysomnography [PSG]) to detect the presence of SDs in PD patients, and the second section focused on cognitive measures, such as the Parkinson Neuropsychometric Dementia Assessment and Test of Attentional Performance. Their main results demonstrated that: (i) in PD patients, insomnia was the most common feature (90%), followed by EDS (89%); and (ii) approximately 70% of PD patients had elevated AHI, 46% suffered from mild-to-moderate OSA, and 27% suffered from moderate OSA. Cognitive performance was decreased in PD-OSA patients, with impairment of executive function/working memory, attention, semantic memory, and processing speed emerging as the most frequent cognitive profile. Interestingly, cognitive dysfunctions correlated with PSG parameters, in particular, sleep quality (reduced sleep efficiency and total sleep time) and the apnea-hypopnea index (AHI >5/h). Despite the small sample size, the added value of this study is the detection of SDs based on objective evaluations, such as PSG recordings.

The third research article is focused on the detection of a specific dopaminergic pattern of neurodegeneration related to RBD in PD patients (Cao et al.). This topic is stimulating. RBD, a parasomnia characterized by the loss of muscle atonia and abnormal behaviors during REM sleep, affects 30–50% of PD patients and can often precede the typical motor manifestation by several years. A specific neurocognitive profile, mainly characterized by a deficit of memory and poorer performance on visuospatial and constructional ability tasks, consequent to the posterior-cortical area has been detected in PD-RBD (Maggi et al., 2021). The contribution of neuroimaging, especially DAT-SPECT imaging, has been substantial in demonstrating the existence of a continuum of the presynaptic nigrostriatal dopaminergic denervation from RBD toward PD pathology. In this context, here, we present a prospective scintigraphic study in a large international multicenter cohort of *de novo* PD, obtained from the PPMI database (Cao et al.). The goal of the study was to test the hypothesis, as the presence of RBD in PD may be associated with a distinct pattern of striatal dopamine denervation detectable by DAT-SPECT imaging. Their imaging protocol included a serial dopaminergic transporter at different timepoints: at baseline and 1, 2, and 4 years after the initial DAT scan. Thus, the authors investigated and compared the striatal DAT binding uptakes and their rates of decline in PD with (PD-RBD) and without RBD. Their main results demonstrated that, in comparison with PD patients without RBD, PD-RBD patients (i) had lower striatal DAT binding in the caudate (which was more pronounced in the less-affected hemisphere) and in the putamen at baseline evaluation, (ii) showed a consistently greater DAT loss during the 4-year follow-up serial evaluations, and (iii) exhibited a more rapid decrease in DAT binding in the caudate (contralateral to clinical manifestations) and a less prominent interhemispheric asymmetry in the putamen. These findings are novel, identifying a caudate degeneration in PD-RBD patients. The results obtained in a large cohort (220 patients) and in drug-naïve PD patients minimize the potential effect of levodopa treatment. Thus, the main contribution of this research article is the identification of a specific scintigraphic pattern of striatal denervation that could be implicated in the pathogenesis of a more severe clinical phenotype.

In conclusion, this Research Topic provides useful information to better define the intriguing PD-SD subtype through the neuroimaging and clinical magnifying glass. Functional imaging and serial DAT-SPECT evaluation contribute to identify the neural circuits underlying the cognitive impairment related to SDs in PD, whereas clinical evaluation is fundamental for characterizing the neurocognitive profile. A frontostriatal trajectory appears to characterize PD cognitive impairment in the presence of EDS and OSA, whereas a posterior-cortical trajectory with caudate degeneration characterizes PD cognitive impairment in the presence of RBD. Emphasis is placed on the early detection of SDs to identify PD patients at high risk of rapid cognitive impairment and develop novel disease-modifying therapies.

## Author contributions

MS contributed to the conception. LF-S contributed to the supervision of the manuscript. Both authors contributed to the article and approved the submitted version.
